# Association of Migraine Headache With Depression, Anxiety, and Stress in the Population of Makkah City, Saudi Arabia: A Cross-Sectional Study

**DOI:** 10.7759/cureus.39788

**Published:** 2023-05-31

**Authors:** Omar Babateen, Fadi S Althobaiti, Muhannad Ahmed Alhazmi, Eyad Al-Ghamdi, Faeqah Alharbi, Alshaymaa K Moffareh, Fay M Matar, Abdullah Tawakul, Jamil A Samkari

**Affiliations:** 1 Department of Physiology, Umm Al-Qura University, Makkah, SAU; 2 Department of Medicine, Umm Al-Qura University, Makkah, SAU; 3 Department of Family and Community Medicine, King Abdulaziz University, Faculty of Medicine, Jeddah, SAU

**Keywords:** anxiety, association, cross sectional, dass-21, depression, ichd-3, makkah city, migraine, saudi arabia, stress

## Abstract

Introduction

Migraine is characterized by persistent headaches and a wide range of symptoms, such as nausea, vomiting, and photophobia. The chance of developing a chronic migraine might be increased by lifestyle variables like obesity, stress, and excessive medication use. According to previous studies in Saudi Arabia, migraines are more common there than they are globally. The study aimed to examine the migraine associations with depression, anxiety, and stress in the population of Makkah City, Saudi Arabia.

Methods

The study employed a descriptive cross-sectional design with a non-probability snowball sampling technique and an online questionnaire that included sociodemographic characteristics, the International Classification of Headache Disorders-3 (ICHD-3) criteria for migraine assessment, and the Depression, Anxiety, and Stress Scale-21 (DASS-21) measure for depression, anxiety, and stress.

Results

Our study included 418 participants, out of whom 73.7% were female and 26.3% were male. Regarding migraine, only 8.9% of participants met the ICHD-3 criteria for migraine headache screening, with a female predominance (78.4%). The study showed a high prevalence of depression, anxiety, and stress among the population (63.9%, 63.6%, and 55%, respectively), with females having a higher prevalence. Depression, anxiety, and stress had an equal prevalence of 78.4% among migraineurs, which was significantly higher than that of non-migraineurs.

Conclusions

The study found significant associations between migraine and depression, anxiety, and stress. This study provides insights into the association between these conditions. The study's findings suggest the need for screening and management of mental health conditions in patients with migraine. However, extensive efforts are needed to be applied in different cities and demographics for a more precise understanding of the association.

## Introduction

Migraine is a primary headache disorder characterized by recurrent attacks of mostly unilateral headaches that are frequently accompanied by nausea, vomiting, and light sensitivity [1]. It is caused by the activation of a deep-brain mechanism that results in the production of pain-inducing inflammatory substances around the head's nerves and blood vessels [2]. Migraines are classified into two types: migraine with aura (MA) and migraine without aura (MO) [3]. Lifestyle-related factors can significantly increase the likelihood of developing migraines and the consequences on the quality of life (QoL). The most important modifiable risk factors for chronic migraine include overuse of acute migraine medication, depression, obesity, and stressful life. In addition, age, female gender, and low educational status are non-modifiable risk factors that increase the risk of chronic migraine [4]. It could be avoided if environmental, nutritional, and behavioral triggers were identified and managed [5].

According to the Global Burden of Disease (GBD), headache disorders are the most prevalent and disabling diseases worldwide. The global prevalence of active headache disorders was 52.0%, of which migraine accounted for 14.0% of these cases [6]. The prevalence of migraine in Saudi Arabia is considerably higher than global averages [7]. A local study has reported the prevalence of migraine headaches to be 37.2%, with a higher prevalence among females (81.1%) and the highest prevalence observed among students (43.3%) [8]. Speaking of mortality, migraine headaches are unlikely to cause death directly. However, due to a higher risk of cardiovascular events, mortality rates were higher in women with migraine with aura [9]. A recent study conducted in Saudi Arabia reported prevalence data for depression, anxiety, and stress among the general population and found a depression prevalence of 28.9%, an anxiety prevalence of 16.4%, and a stress prevalence of 11.9% [10].

In a cross-sectional observational study conducted by Pearl et al. on 567 predominantly female (87.3%) migraine patients, they found a positive correlation between the patient’s Migraine Disability Assessment Scale (MIDAS) and their Patient Health Questionnaire 2 (PHQ-2) [11]. AlQarni et al. conducted a descriptive cross-sectional survey in the Aseer region of Saudi Arabia on 1123 adults, of whom 152 (13.5%) reported no headache, 833 (74.2%) had non-migraine headaches (NMH), and 138 (12.3%) had migraine headaches, depression was reported in 26.1% of migraine patients, compared to 10.9% and 6.6% in NMH cases and adults with no headache, respectively [12].

Another study, conducted in Saudi Arabia, assessed 247 migraine patients aged between 16 and 45 years using the Depression Anxiety Stress Scale (DASS-21) questionnaire and found that 73.3% of the patients met the criteria for anxiety, while 70.9% and 72.3% of patients met the criteria for depression and stress, respectively [13]. Furthermore, a study on 1340 female students at Taif University in Saudi Arabia found that 32.5% of them have migraines and report the main triggers for migraines and stress and anxiety. The study also reported that 51.8% of migraine students were depressed [14]. Additionally, a study conducted in 2012 that aimed to assess the role of depression in migraine chronification concluded that depression is a significant predictor of migraine chronicity [15].

As per the author's knowledge, this was the first study conducted to assess the relationship between migraine headaches and anxiety, depression, and stress in Makkah City, Saudi Arabia, while only a few studies have been conducted worldwide. Therefore, the study aimed to increase understanding of the association of migraine headaches with depression, anxiety, and stress among the population of Makkah City, Saudi Arabia, and to develop more effective strategies for managing these conditions in this specific cultural and environmental context.

## Materials and methods

Study design

The study employed a descriptive cross-sectional study design with a non-probability snowball sampling technique as the sampling method. An online questionnaire (in Arabic) consisting of three parts was used for sociodemographic characteristics (Appendix). In addition, the International Classification of Headache Disorders (ICHD-3) criteria for migraine assessment and the DASS-21 measured the association between depression, anxiety, and stress in migraine patients [16,17]. The questionnaire was transferred to Google Forms and administered electronically to participants via social media platforms.

Study population

The study's target population was general Arabic and English-speaking adults living in Makkah, Saudi Arabia. This study excluded participants who failed to complete the questionnaire, lived outside of Makkah City, and spoke neither Arabic nor English language.

Sampling methodology

The survey was conducted among the general public in Makkah City, Saudi Arabia, from February 26 to April 1, 2023. A written consent form was obtained from all participants before they filled out the questionnaire. According to OpenEpi version 3.1, a sample size of at least 384 participants was considered for a confidence interval level of 95%, an anticipated percentage of frequency of 50%, and a design effect of 1. The questionnaire consisted of three parts and was written in Arabic and English languages. The first part of the questionnaire included sociodemographic characteristics, such as age, gender, nationality, marital status, level of education, occupation, and income. The second part consisted of ICHD-3 migraine criteria. Following that, participants were asked to complete the third part of the questionnaire, which consists of DASS-21 to assess the presence of depression, anxiety, and stress. The anonymity of survey respondents was maintained, and their personal information, such as name, address, phone number, or email address, was not collected in electronic data collection forms; the data were automatically entered into an Excel spreadsheet. After verification and filtering, the data were transferred to BlueSky Statistics version 10.2.1 (Chicago, IL: BlueSky Statistics LLC) for analysis.

Data analysis

Data were extracted, reviewed, coded, and entered into BlueSky Statistics version 10.2.1 statistical software. The results were presented as frequencies and percentages. Descriptive statistics were obtained for all sociodemographic variables, including participants' age in years, gender, nationality, marital status, education level, occupation, and family monthly income in Saudi Riyal (SAR), and analysis based on frequency and percent distribution was performed for these sociodemographic variables. Migraine prevalence was estimated using ICHD-3 criteria, and the symptoms' frequencies and percentages were plotted in a graph. As for DASS-21, frequency and percentage were tabulated for depression, anxiety, and stress with the following different levels: normal, mild, moderate, severe, and extremely severe for all participants. The chi-square test was used to assess the association between demographics and migraine, demographics and depression, anxiety and stress, and lastly, depression, anxiety, and stress with migraine. A significant association was determined by a p-value of <0.05.

Ethical part and confidentiality

Consent was obtained from each participant through the questionnaire. The aims of the research were stated in the questionnaire form for all participants. Additionally, all participants' identities were kept anonymous and confidential. The responses were only accessible to the investigators. Ethical approval was obtained from the Biomedical Ethics Committee of Umm Alqura University (UQU) (#HAPO-02-K-012-2023-02-1476).

## Results

A total of 567 individuals participated in the study. One participant was excluded considering he was under the age of 18 years, and 148 were excluded because they lived outside of Makkah City, leaving the included sample size at 418. The sociodemographic data in Table 1 show that the sample is predominantly female - 308 (73.7%), with only 110 (26.3%) males. The participants’ ages ranged from 18 to 55 years, with the majority of the participants ranging in age from 18 to 25 years (46.9%). A total of 393 participants were Saudis (94%), of which 318 had a university-level education (76.1%). Around 50.2% of them were single, and 46.2% were married.

**Table 1:** Sociodemographic characteristics of participants.

Variables		Demographic data	
		n=418	%
Gender	Male	110	26.3%
	Female	308	73.7%
Age (years)	18-25	196	46.9%
	26-35	38	9.1%
	36-45	71	17.0%
	46-55	88	21.1%
	>55	25	6.0%
Nationality	Saudi	393	94.0%
	Non-Saudi	25	6.0%
Marital status	Single	210	50.2%
	Married	193	46.2%
	Divorced	10	2.4%
	Widowed	5	1.2%
Education level	Primary	1	0.2%
	Intermediate	5	1.2%
	Secondary	66	15.8%
	University	318	76.1%
	Postgraduate	28	6.7%
Occupation	Student	181	43.3%
	Professional	149	35.6%
	Self-employed	17	4.1%
	Retired	25	6.0%
	Housewife	37	8.9%
	Unemployed	9	2.2%
Family monthly income	0-5,000	84	20.1%
	5,001-10,000	86	20.6%
	10,001-15,000	99	23.7%
	15,000-20,000	81	19.4%
	>20,000	68	16.3%

**Figure 1:**
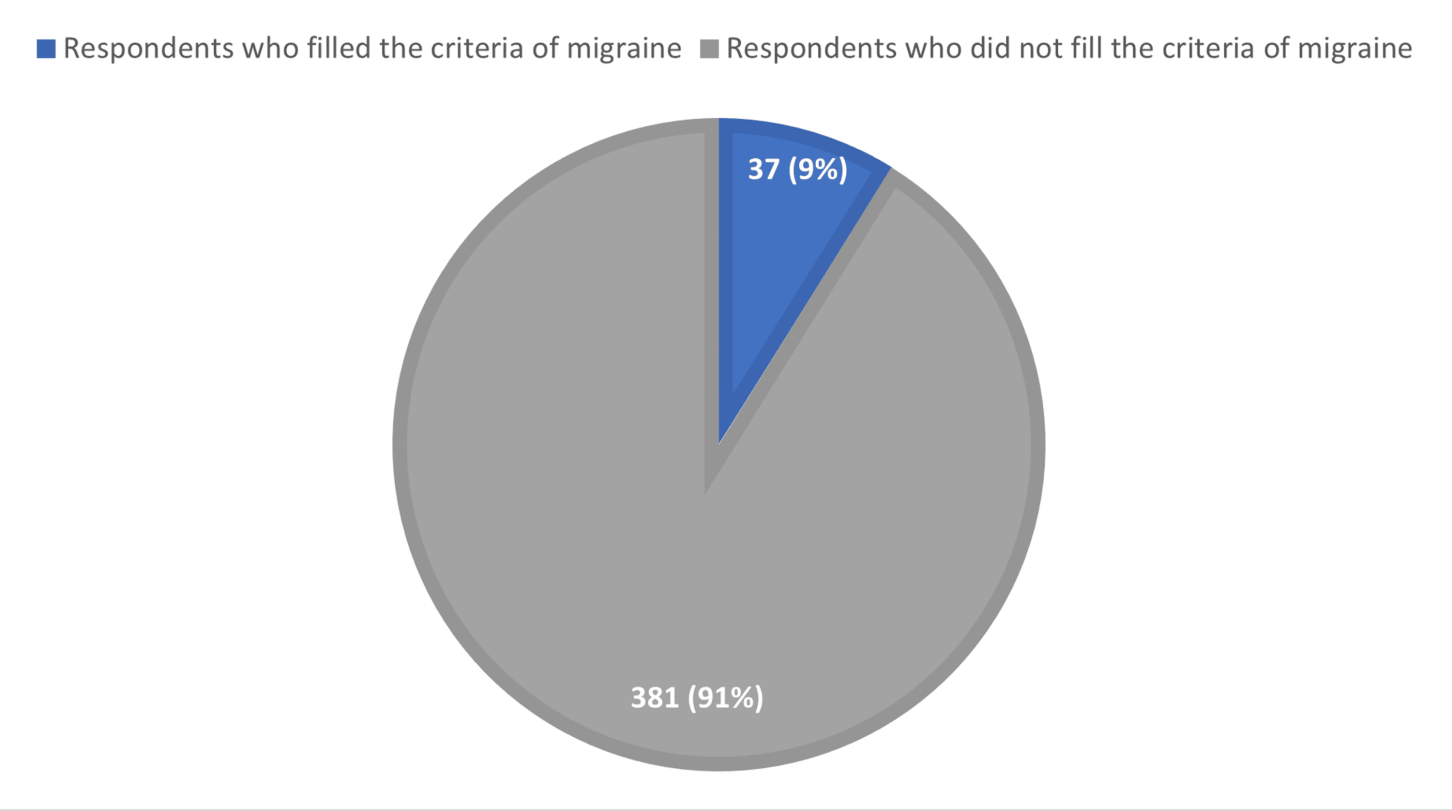
Prevalence of migraine in participants.

**Figure 2:**
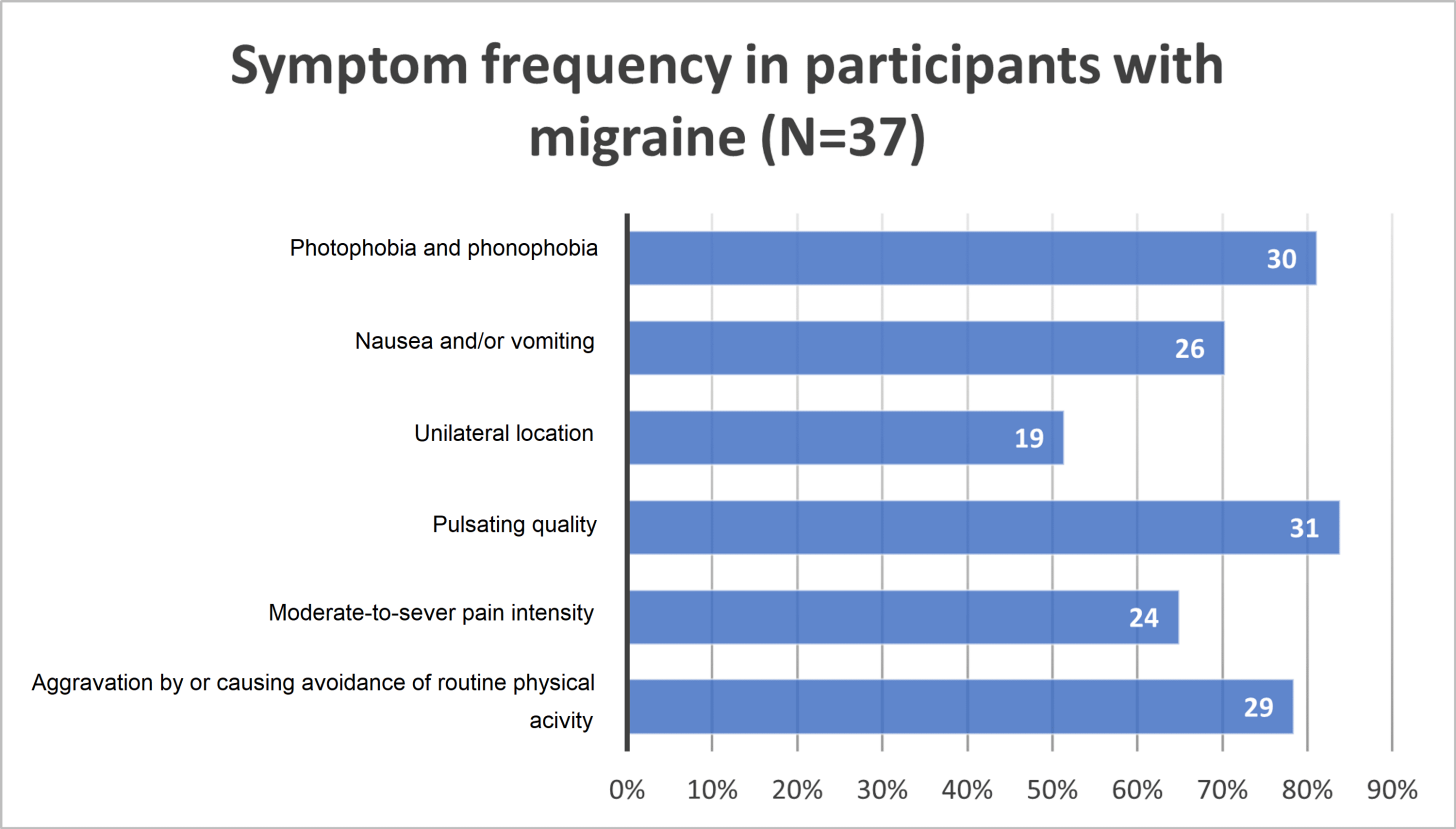
Frequency of migraine symptoms.

Figure 1 shows that only 37 (8.9%) of the 418 participants met the ICHD-3 criteria for migraine headache screening. A pulsating headache was the most frequently reported symptom, as shown in Figure 2, followed by photophobia and phonophobia (83.8% and 81.1%), followed by nausea and/or vomiting at 70.3%. Moderate-to-severe headaches were reported in 64.9% of participants, and 50% of them reported that their headache was unilateral.

Depression prevalence results are presented in Table 2. It indicates that approximately a third of the 151 participants (36.1%) showed no signs of depression. A total of 50 participants (12%) reported mild depression, which is followed by moderate depression, reported in 82 (19.6%), severe depression in 49 (11.7%), and extremely severe depression in 86 (20.6%). In terms of anxiety, 152 (36.4%) participants were unaffected by anxiety, while 28 (6.7%) had mild anxiety, 80 (19.1%) had moderate anxiety, 47 (11.2%) complained of severe anxiety, and lastly, an alarming 111 (26.5%) participants suffered from extremely severe anxiety. In the context of stress, 188 (45%) were normal. A total of 46 (11%) participants had mild stress, 70 (16.7%) complained of moderate stress, 62 (14.8%) suffered from severe stress, and 52 (12.4%) reported extremely severe levels of stress.

**Table 2:** Prevalence of depression, anxiety, and stress.

Variables		n=418	%
Depression	Normal	151	36.1%
	Mild	50	12.0%
	Moderate	82	19.6%
	Severe	49	11.7%
	Extremely severe	86	20.6%
Anxiety	Normal	152	36.4%
	Mild	28	6.7%
	Moderate	80	19.1%
	Severe	47	11.2%
	Extremely severe	111	26.5%
Stress	Normal	188	45.0%
	Mild	46	11.0%
	Moderate	70	16.7%
	Severe	62	14.8%
	Extremely severe	52	12.4%

Table 3 shows that females had a higher prevalence of migraine than males, with 29 (9.4%) females and eight (7.3%) males. The prevalence rates for age groups 36-45 and 18-25 were eight (11.3%) and 22 (11.2%), respectively. Participants aged 46-55 years scored the lowest prevalence of migraine at 3.4%. No sociodemographic variable was significantly associated with migraine.

**Table 3:** Association between migraine headache and sociodemographic variables.

Variables		Without migraine		With migraine		p-Value
		n	%	n	%	
Gender	Male	102	92.7%	8	7.3%	0.5
	Female	279	90.6%	29	9.4%	
Age (years)	18-25	174	88.8%	22	11.2%	0.2
	26-35	35	92.1%	3	7.9%	
	36-45	63	88.7%	8	11.3%	
	46-55	85	96.6%	3	3.4%	
	>55	24	96.0%	1	4.0%	
Nationality	Saudi	358	91.1%	35	8.9%	0.9
	Non-Saudi	23	92.0%	2	8.0%	
Marital status	Single	187	89.0%	23	11.0%	0.4
	Married	180	93.3%	13	6.7%	
	Divorced	9	90.0%	1	10.0%	
	Widowed	5	100%	0	0%	
Education level	Primary	1	100%	0	0%	0.6
	Intermediate	5	100%	0	0%	
	Secondary	63	95.5%	3	4.5%	
	University	287	90.3%	31	9.7%	
	Postgraduate	25	89.3%	3	10.7%	
Occupation	Student	161	89.0%	20	11.0%	0.4
	Professional	135	90.6%	14	9.4%	
	Self-employed	17	100%	0	0%	
	Retired	24	96.0%	1	4.0%	
	Housewife	36	97.3%	1	2.7%	
	Unemployed	8	88.9%	1	11.1%	
Family monthly income	0-5,000	78	92.9	6	7.1%	0.6
	5,001-10,000	76	88.4%	10	11.6%	
	10,001-15,000	88	88.9%	11	11.1%	
	15,000-20,000	76	93.8%	5	6.2%	
	>20,000	63	92.4%	5	7.4%	

As shown in Table 4, females had a depression prevalence rate of 67.3% (207), of which the mild, moderate, severe, and extremely severe depression prevalence rates were 11.7% (36), 21.1% (65), 10.1% (31), and 24.4% (75), respectively. Depression prevalence was highest among those aged 18-25 years (71.4%), followed by adults aged 26-35 years (68.4%). Participants aged 55 years and above had the lowest prevalence of depression (36%). Single participants showed a higher prevalence of depression (72.9%), followed by divorced participants (70%) when compared to married participants (54.9%). Participants with family monthly income of 0-5,000 (SAR) scored the highest prevalence of depression at 76.2%, followed by participants with family monthly income of >20,000 (SAR) and 15,001-20,000 (SAR) at 64.7% and 64.2%, respectively, while the lowest rate of prevalence was among those with family monthly income of 10,001-15,000 (SAR) at 54.5%. The only significant variables in the association between depression and sociodemographic data were gender (p=0.003), marital status (p=0.033), and monthly family income (p=0.0049).

**Table 4:** Association between depression and sociodemographic variables. *P-value<0.01 is statistically highly significant. **P-value<0.05 is statistically significant.

Variables			Depression scale					p-Value
			Normal	Mild	Moderate	Severe	Extremely severe	
Gender	Female	n	101	36	65	31	75	0.003*
		%	32.8%	11.7%	21.1%	10.1%	24.4%	
	Male	n	50	14	17	18	11	
		%	45.5%	12.7%	15.5%	16.4%	10.0%	
Age (years)	>55	n	16	1	5	1	2	0.18
		%	64.0%	4.0%	20.0%	4.0%	8.0%	
	18-25	n	56	26	38	28	48	
		%	28.6%	13.3%	19.4%	14.3%	24.5%	
	26-35	n	12	4	8	5	9	
		%	31.6%	10.5%	21.1%	13.2%	23.7%	
	36-45	n	29	8	15	5	14	
		%	40.8%	11.3%	21.1%	7.0%	19.7%	
	46-55	n	38	11	16	10	13	
		%	43.2%	12.5%	18.2%	11.4%	14.8%	
Nationality	Non-Saudi	n	8	3	5	4	5	0.97
		%	32.0%	12.0%	20.0%	16.0%	20.0%	
	Saudi	n	143	47	77	45	81	
		%	36.4%	12.0%	19.6%	11.5%	20.6%	
	Total	n	151	50	82	49	86	
Marital status	Divorced	n	3	1	1	2	3	0.033**
		%	30.0%	10.0%	10.0%	20.0%	30.0%	
	Married	n	87	21	37	18	30	
		%	45.1%	10.9%	19.2%	9.3%	15.5%	
	Single	n	57	28	43	29	53	
		%	27.1%	13.3%	20.5%	13.8%	25.2%	
	Widowed	n	4	0	1	0	0	
		%	80.0%	0	20.0%	0	0	
	Total	n	151	50	82	49	86	
Educational level	Intermediate	n	2	1	1	0	1	0.49
		%	40.0%	20.0%	20.0%	0	20.0%	
	Postgraduate	n	12	1	7	3	5	
		%	42.9%	3.6%	25.0%	10.7%	17.9%	
	Primary	n	0	0	0	1	0	
		%	0	0	0	100.0%	0	
	Secondary	n	17	8	17	8	16	
		%	25.8%	12.1%	25.8%	12.1%	24.2%	
	University	n	120	40	57	37	64	
		%	37.7%	12.6%	17.9%	11.6%	20.1%	
	Total	n	151	50	82	49	86	
Occupation	Housewife	n	15	3	5	5	9	0.12
		%	40.5%	8.1%	13.5%	13.5%	24.3%	
	Professional	n	62	17	34	12	24	
		%	41.6%	11.4%	22.8%	8.1%	16.1%	
	Retired	n	15	0	3	3	4	
		%	60.0%	0	12.0%	12.0%	16.0%	
	Self-employed	n	3	4	3	4	3	
		%	17.6%	23.5%	17.6%	23.5%	17.6%	
	Student	n	53	26	34	24	44	
		%	29.3%	14.4%	18.8%	13.3%	24.3%	
	Unemployed	n	3	0	3	1	2	
		%	33.3%	0.0	33.3%	11.1%	22.2%	
Family monthly income	>20,000	n	24	6	14	12	12	0.0049*
		%	35.3%	8.8%	20.6%	17.6%	17.6%	
	0-5,000	n	20	11	28	8	17	
		%	23.8%	13.1%	33.3%	9.5%	20.2%	
	10,001-15,000	n	45	11	14	4	25	
		%	45.5%	11.1%	14.1%	4.0%	25.3%	
	15,000-20,000	n	29	11	10	17	14	
		%	35.8%	13.6%	12.3%	21.0%	17.3%	
	5001-10000	n	33	11	16	8	18	
		%	38.4%	12.8%	18.6%	9.3%	20.9%	

Table 5 shows that 212 (68.8%) female participants reported anxiety, with 20 (6.5%) suffering from mild anxiety, 62 (20.1%) moderate anxiety, 37 (12%) severe anxiety, and 93 (30.2%) suffering from extremely severe anxiety. Participants aged 26-35 years scored the highest levels of anxiety (71.1%), while participants over the age of 55 years scored the lowest prevalence of anxiety (44%). A total of 251 (63.9%) Saudi participants were affected by anxiety compared to non-Saudi participants, who had a prevalence of 15 (60%). Gender was the only sociodemographic variable with a significant association (p=0.0033) with anxiety.

**Table 5:** Association between anxiety and sociodemographic variables. *P-value<0.01 is statistically highly significant.

Variables			Anxiety scale					p-Value
			Normal	Mild	Moderate	Severe	Extremely severe	
Gender	Female	n	96	20	62	37	93	0.0033*
		%	31.2%	6.5%	20.1%	12.0%	30.2%	
	Male	n	56	8	18	10	18	
		%	50.9%	7.3%	16.4%	9.1%	16.4%	
Age (years)	>55	n	14	1	4	3	3	0.24
		%	56.0%	4.0%	16.0%	12.0%	12.0%	
	18-25	n	58	13	46	23	56	
		%	29.6%	6.6%	23.5%	11.7%	28.6%	
	26-35	n	11	2	6	7	12	
		%	28.9%	5.3%	15.8%	18.4%	31.6%	
	36-45	n	31	4	9	6	21	
		%	43.7%	5.6%	12.7%	8.5%	29.6%	
	46-55	n	38	8	15	8	19	
		%	43.2%	9.1%	17.0%	9.1%	21.6%	
Nationality	Non-Saudi	n	10	1	4	4	6	0.89
		%	40.0%	4.0%	16.0%	16.0%	24.0%	
	Saudi	n	142	27	76	43	105	
		%	36.1%	6.9%	19.3%	10.9%	26.7%	
Marital status	Divorced	n	4	0	1	0	5	0.138
		%	40.0%	0	10.0%	0	50.0%	
	Married	n	85	13	30	20	45	
		%	44.0%	6.7%	15.5%	10.4%	23.3%	
	Single	n	60	15	48	27	60	
		%	28.6%	7.1%	22.9%	12.9%	28.6%	
	Widowed	n	3	0	1	0	1	
		%	60.0%	0	20.0%	0	20.0%	
Educational level	Intermediate	n	1	1	2	0	1	0.577
		%	20.0%	20.0%	40.0%	0	20.0%	
	Postgraduate	n	13	1	5	3	6	
		%	46.4%	3.6%	17.9%	10.7%	21.4%	
	Primary	n	0	0	1	0	0	
		%	0	0	100.0%	0	0	
	Secondary	n	20	3	11	12	20	
		%	30.3%	4.5%	16.7%	18.2%	30.3%	
	University	n	118	23	61	32	84	
		%	37.1%	7.2%	19.2%	10.1%	26.4%	
Occupation	Housewife	n	14	1	5	5	12	0.594
		%	37.8%	2.7%	13.5%	13.5%	32.4%	
	Professional	n	63	10	23	15	38	
		%	42.3%	6.7%	15.4%	10.1%	25.5%	
	Retired	n	12	2	2	5	4	
		%	48.0%	8.0%	8.0%	20.0%	16.0%	
	Self-employed	n	5	1	4	3	4	
		%	29.4%	5.9%	23.5%	17.6%	23.5%	
	Student	n	54	13	45	18	51	
		%	29.8%	7.2%	24.9%	9.9%	28.2%	
	Unemployed	n	4	1	1	1	2	
		%	44.4%	11.1%	11.1%	11.1%	22.2%	
Family monthly income	>20,000	n	28	3	12	7	18	0.73
		%	41.2%	4.4%	17.6%	10.3%	26.5%	
	0-5,000	n	26	3	16	13	26	
		%	31.0%	3.6%	19.0%	15.5%	31.0%	
	10,001-15,000	n	35	6	24	11	23	
		%	35.4%	6.1%	24.2%	11.1%	23.2%	
	15,000-20,000	n	30	10	14	8	19	
		%	37.0%	12.3%	17.3%	9.9%	23.5%	
	5,001-10,000	n	33	6	14	8	25	
		%	38.4%	7.0%	16.3%	9.3%	29.1%	

The prevalence of stress is shown in Table 6 for females at 180 (58.4%) and 50 (45.5%) for males. Participants aged 18-25 years had the highest prevalence out of all the age groups at 123 (65.8%), while participants over 55 years had the lowest prevalence at six (24%). Divorced participants had a higher prevalence (70%) when compared to married and single participants, 85 (44%), and 137 (65.2%), respectively. Out of all the demographic data, only gender (p=0.0087) and marital status (p=0.0074) had significant associations with stress.

**Table 6:** Association between stress and sociodemographic variables. *P-value<0.01 is statistically highly significant.

Variables			Stress scale					p-Value
			Normal	Mild	Moderate	Severe	Extremely Severe	
Gender	Female	n	128	35	47	53	45	0.0087*
		%	41.6%	11.4%	15.3%	17.2%	14.6%	
	Male	n	60	11	23	9	7	
		%	54.5%	10.0%	20.9%	8.2%	6.4%	
Age (years)	>55	n	19	1	3	0	2	0.074
		%	76.0%	4.0%	12.0%	0	8.0%	
	18-25	n	73	25	38	32	28	
		%	37.2%	12.8%	19.4%	16.3%	14.3%	
	26-35	n	13	5	7	9	4	
		%	34.2%	13.2%	18.4%	23.7%	10.5%	
	36-45	n	36	8	7	11	9	
		%	50.7%	11.3%	9.9%	15.5%	12.7%	
	46-55	n	47	7	15	10	9	
		%	53.4%	8.0%	17.0%	11.4%	10.2%	
Nationality	Non-Saudi	n	14	3	2	2	4	0.55
		%	56.0%	12.0%	8.0%	8.0%	16.0%	
	Saudi	n	174	43	68	60	48	
		%	44.3%	10.9%	17.3%	15.3%	12.2%	
Marital status	Divorced	n	3	0	3	1	3	0.0074*
		%	30.0%	0	30.0%	10.0%	30.0%	
	Married	n	108	17	26	23	19	
		%	56.0%	8.8%	13.5%	11.9%	9.8%	
	Single	n	73	29	40	38	30	
		%	34.8%	13.8%	19.0%	18.1%	14.3%	
	Widowed	n	4	0	1	0	0	
		%	80.0%	0	20.0%	0	0	
Educational Level	Intermediate	n	2	1	0	1	1	0.353
		%	40.0%	20.0%	0	20.0%	20.0%	
	Postgraduate	n	12	3	5	3	5	
		%	42.9%	10.7%	17.9%	10.7%	17.9%	
	Primary	n	0	0	1	0	0	
		%	0	0	100.0%	0	0	
	Secondary	n	25	10	17	11	3	
		%	37.9%	15.2%	25.8%	16.7%	4.5%	
	University	n	149	32	47	47	43	
		%	46.9%	10.1%	14.8%	14.8%	13.5%	
Occupation	Housewife	n	15	6	7	6	3	0.2
		%	40.5%	16.2%	18.9%	16.2%	8.1%	
	Professional	n	83	11	19	19	17	
		%	55.7%	7.4%	12.8%	12.8%	11.4%	
	Retired	n	13	2	6	2	2	
		%	52.0%	8.0%	24.0%	8.0%	8.0%	
	Self-employed	n	7	1	5	1	3	
		%	41.2%	5.9%	29.4%	5.9%	17.6%	
	Student	n	67	25	30	34	25	
		%	37.0%	13.8%	16.6%	18.8%	13.8%	
	Unemployed	n	3	1	3	0	2	
		%	33.3%	11.1%	33.3%	0	22.2%	
Family monthly income	>20,000	n	25	7	16	12	8	0.38
		%	36.8%	10.3%	23.5%	17.6%	11.8%	
	0-5,000	n	34	12	15	12	11	
		%	40.5%	14.3%	17.9%	14.3%	13.1%	
	10,001-15,000	n	51	8	14	10	16	
		%	51.5%	8.1%	14.1%	10.1%	16.2%	
	15,000-20,000	n	39	5	16	13	8	
		%	48.1%	6.2%	19.8%	16.0%	9.9%	
	5,001-10000	n	39	14	9	15	9	
		%	45.3%	16.3%	10.5%	17.4%	10.5%	

As indicated in Table 7, the prevalence of mild, moderate, severe, and extremely severe depression in respondents suffering from migraines was two (5.4%), eight (21.6%), five (13.5%), and 14 (37.8%), respectively, when compared to non-migraineurs, who had a prevalence of 48 (12.6%), 74 (19.4%), 44 (11.5%), and 72 (18.9%), respectively. When it comes to anxiety, migraineurs reported no mild anxiety but a higher prevalence of moderate anxiety (5, 13.5%), severe anxiety (3, 8.1%), and extremely severe anxiety (21, 56.8%), when compared to non-migraineurs, they reported a prevalence of mild anxiety (28, 7.3%), moderate anxiety (75, 19.7%), severe anxiety (44, 11.5%), and extremely severe anxiety (90, 23.6%). Finally, there was an increased prevalence of stress among migraineurs, with three (8.1%) reporting mild stress, eight (21.6%) having moderate stress, four (10.8%) reporting severe stress, and 14 (37.8%) reporting extremely severe stress, where non-migraineurs reported mild, moderate, severe, and extremely severe stress of 43 (11.3%), 62 (16.3%), 58 (15.2%), and 38 (10%). Depression, anxiety, and stress were all found to be significantly associated with migraines (p=0.04, p=0.0005, and p=0.00002, respectively).

**Table 7:** Association between migraine and depression, anxiety, and stress. *P-value<0.05 is statistically significant. **P-value<0.01 is statistically highly significant.

Variables			Depression scale					p-Value
			Normal	Mild depression	Moderate depression	Severe depression	Extremely severe depression	
Migraine status	Migraine	n	8	2	8	5	14	0.04*
		%	21.6%	5.4%	21.6%	13.5%	37.8%	
	Non-migraine	n	143	48	74	44	72	
		%	37.5%	12.6%	19.4%	11.5%	18.9%	
	Migraine	n	8	0	5	3	21	0.0005**
		%	21.6%	0	13.5%	8.1%	56.8%	
	Non-migraine	n	144	28	75	44	90	
		%	37.8%	7.3%	19.7%	11.5%	23.6%	
	Migraine	n	8	3	8	4	14	0.00002**
		%	21.6%	8.1%	21.6%	10.8%	37.8%	
	Non-migraine	n	180	43	62	58	38	
		%	47.2%	11.3%	16.3%	15.2%	10.0%	

## Discussion

The purpose of this cross-sectional study was to determine the relationship between migraine headaches and depression, anxiety, and stress in the Makkah City, Saudi Arabia, population. Our study findings revealed that only 8.9% of the study participants reported experiencing migraines. The study's migraine prevalence is lower than the global prevalence of 14% [6]. This contradicts the findings of a previous study, which concluded that migraine prevalence in Saudi Arabia is higher than the global average [7]. The most reported symptom was a pulsating headache, followed by photophobia, phonophobia, nausea, and vomiting. A higher prevalence result was reported for the female gender, which was also consistent with findings reported in a previously published study [11-13]. This gender difference can be explained by the fluctuations in estrogen and progesterone, which have been associated with migraine pathogenesis [18].

The number of participants who reported normal scores regarding depression was 151 (36.1%), 152 (36.4%), and 188 (45%) for anxiety and stress. A larger proportion of participants (63.9%) reported mild, moderate, severe, or extremely severe depression, which is also found to be an alarming increase in prevalence when compared with a previous 2021 study conducted in the city of Jeddah, Saudi Arabia, studying the prevalence of migraine and its effect on QoL among the general population that concluded a depression prevalence of 37.2% [8]. In the current study, the reports of mild, moderate, severe, or extremely severe anxiety were (63.6%), and the reports of mild, moderate, severe, and extremely severe stress were (55%). The results indicate an increase in prevalence when compared to the findings of a recent 2020 study in Saudi Arabia that aimed to study the prevalence of depression, anxiety, and stress among the general population and found an anxiety prevalence of 16.4% and a stress prevalence of 11.9%. This difference could be attributed to the variation in sample demographic characteristics [10]. The findings of the current study reported that gender was significantly associated with depression, anxiety, and stress, with females being more susceptible to all of them. Additionally, depression and stress were strongly associated with marital status, with singles and divorcees being more susceptible. Despite female susceptibility to depression, anxiety, and stress, a recent 2021 study in the region demonstrated an insignificant association between gender and marital status (p>0.05) [13].

Our study found that depression, anxiety, and stress were all significantly associated with migraine, with an equally high prevalence of 78.4% among migraineurs. This is consistent with a previous study conducted among migraine patients in Saudi Arabia, which found abnormal scores for depression (70.9%), anxiety (73.3%), and stress (72.3%) [13]. Another 2020 study conducted in North America studied the impact of depression and anxiety symptoms in migraineurs and found a corresponding percentage of anxiety prevalence (75.3%), but the prevalence of depression was significantly lower (18%) [11].

The bidirectional relationship between depression and migraine has been observed in previous studies, with depression being a strong predictor of the progression of migraine [15]. However, the exact mechanism underlying this association is unclear, but one hypothesis suggests that it may be due to low levels of 5-hydroxytryptamine (5-HT) or serotonin receptors [19]. As for anxiety, a study conducted in Taif City reported anxiety as one of the main triggers for migraine attacks [14]. Another study conducted in Jeddah also found that stress and anxiety accounted for 81.6% of the observed triggering factors for migraines [8]. Furthermore, stress was identified as a migraine trigger in the Taif City study [14].

The concurrent presence of depression, anxiety, and stress with migraine can significantly affect the QoL of affected individuals. We recommend screening patients with migraines for the presence or development of these mental health conditions, as well as the need to manage them effectively. It is critical to prevent the development of anxiety disorders and depression in migraine patients, which can be accomplished by reducing the number of headache episodes with effective prophylactic pharmacotherapy. We also recommend that further studies be conducted to better understand the relationship between migraine and these mental health disorders in order to develop a more coordinated and direct approach to aid in the diagnosis and management of these conditions.

Limitations

Despite making an effort to obtain accurate, precise, and representative outcomes, this study encountered certain limitations. Firstly, collecting data using an online questionnaire has inherent limitations. Second, the majority of the respondents were female (73.7%), Saudi (94.0%), and had a university-level education (76.1%). Another limitation is that the migraine prevalence in the study sample is relatively small (8.9%). These factors may have resulted in unintended biases in the findings. It is important to consider these limitations when interpreting the results of the study and to use caution when generalizing the findings to other populations or contexts. Nonetheless, this study provides insight into the current situation and confirms the outcomes of previous global and local research on the subject. The study used standardized measures to assess migraine headaches, depression, anxiety, and stress, which increases the reliability and validity of the study findings. More research in this area is required, using different methodologies, focusing on different sociodemographic characteristics, and exploring other unexplored regions of Saudi Arabia.

## Conclusions

Our study provides valuable insights into the association between migraine and depression, anxiety, and stress in the population of Makkah City. The findings highlight the importance of screening and effective management of mental health conditions in patients with migraine to improve their QoL, as the results showed a significant association between migraine and depression, anxiety, and stress. Further studies with a larger sample are warranted in different cities and demographics to better understand the relationship between these conditions and develop more effective interventions.

## Appendices

علاقة الصداع النصفي مع الاكتئاب والقلق والتوتر بين البالغين في مدينة مكة المكرمة، المملكة العربية السعودية: دراسة مقطعية.

Association of Migraine Headache with Depression, Anxiety, and Stress in the Population of Makkah City, Saudi Arabia: A Cross-Sectional Study (English translation)

ندعوكم للمشاركة في هذا الاستبيان المقدم من فريق بحثي يهدف الى قياس علاقة الشقيقة وارتباطها مع الإكتئاب والقلق والتوتر عند البالغين في مدينة مكة المكرمة في المملكة العربية السعودية. المشاركة في هذه الدراسة تطوعية و جميع المعلومات سيتم التعامل معها بكامل السرية واستخدامها لأهداف بحثية فقط. لن يتم نشر أي معلومات شخصية، في حال الموافقة للمشاركة في هذا الاستيبان الرجاء إكماله وارساله عند الإنتهاء منه. شاكرين حسن تعاونكم.

This questionnaire is conducted by a research team that aims to measure the association of migraine headache with depression, anxiety, and stress in Makkah City. All information will be treated with complete confidentiality and used for research purposes only. No personal information will be published. If you agree to participate in our study, please complete and send the following questionnaire. Thank you for your contribution (English translation).

**Table 8:** Sociodemographic characteristics.

Question	Answers																			
الجنس Gender	ذكر Male										أنثى Female									
العمر Age (years)	<18	18-25					26-35				36-45				46-55				>55	
الجنسية Nationality	سعودي Saudi										غير سعودي Non-Saudi									
مدينة السكن City of residence	مكة المكرمة Makkah										أخرى Other									
الحالة الإجتماعية Marital status	أعزب Single					متزوج Married					مطلق Divorced						أرمل Widowed			
المستوى الدراسي Education level	ابتدائي Primary			متوسط Intermediate					ثانوي Secondary				جامعي University					دراسات عليا Postgraduate		
الوظيفة Occupation	طالب Student		موظف Professional					مهنة حرة Self-employed				متقاعد Retired				ربة منزل Housewife				غير موظف Unemployed
دخل العائلة الشهري (بالريال السعودي) Family monthly income (In Saudi Riyal)	0-5,000				5,001-10,000					10,001-15,000				15,001-20,000					>20,000	

**Table 9:** ICHD-3 criteria for migraine without aura. ICHD-3: International Classification of Headache Disorders-3

Question	Answers									
عادة ما أواجه نوبات صداع تستمر بين ٤-٧٢ ساعة عند عدم علاجها؟ I have experienced headache attacks lasting 4-72 hours (untreated or unsuccessfully treated).	Yes					No				
كم مرة واجهت هذه النوبة؟ How many times have you experienced the attacks?	أقل من ٥ مرات Less than 5 attacks		أكثر من ٥ مرات More than 5 attacks					لم أمر بهذه النوبة من قبل I have not experienced any attacks		
خصائص الصداع الذي يواجهني هي كالتالي (تستطيع إختيار أكثر من إجابة) Headache is characterized by (you can choose multiple answers)	صداع مقتصر على منتصف الرأس Unilateral location	طبيعة الصداع نابضة Pulsating quality			أستطيع وصف شدة الألم من متوسطة إلى شديدة Moderate-to-severe pain intensity		تزداد حدة الصداع مع ممارسة النشاطات البدنية مما يؤدي إلى تجنبها في فترة نوبة الصداع Aggravation by or causing avoidance of routine physical activity			لا أواجه أي مما سبق None of the above
أواجه الأعراض التالية أثناء نوبة الصداع During headache attacks, I have experienced	غثيان أو إستفراغ Nausea and/or vomiting			حساسية تجاه الأصوات أو الإضاءة Photophobia and phonophobia					لا أواجه أي مما سبق None of the above	
كم مرة واجهت أحد الأعراض السابقة أثناء نوبة الصداع؟ How many times have you experienced the previous symptoms during a headache attack?	أقل من ٥ مرات Less than 5 attacks			أكثر من ٥ مرات More than 5 attacks					لم أمر بهذه النوبة من قبل I have not experienced any symptoms	

مقياس الإكتئاب، والقلق، والتوتر:

أقرأ كل من النصوص التالية ثم ضع علامة على الرقم (٠، ١، ٢، أو ٣) الذي يبين درجة إنطباق هذا الشعور عليك في الأسبوع الماضي، لا يوجد إجابات صحيحة أو خاطئة، لا تقضي وقتًا طويلًا في أي منها.

استعمل التقديرات التالية عند الإجابة:

٠) لا ينطبق علي بتاتًا.

١) ينطبق علي بعض الشيء أو قليلًا من الأوقات.

٢) ينطبق علي بدرجة ملحوظة أو بعض الأوقات.

٣) ينطبق علي كثيرًا جدًا، أو معظم الأوقات.

DASS-21 questionnaire (English translation):

Please read each statement and circle a number (0, 1, 2, or 3) that indicates how much the statement applied to you over the past week. There are no right or wrong answers. Do not spend too much time on any statement.

The rating scale is as follows: 0 - did not apply to me at all. 1 - Applied to me to some degree, or some of the time. 2 - Applied to me to a considerable degree or a good part of time. 3 - Applied to me very much or most of the time.

**Table 10:** Depression, Anxiety, and Stress Scale-21 (DASS-21) questionnaire.

وجدت صعوبة في الاسترخاء والراحة. I found it hard to wind down.	0	1	2	3
شعرت بجفاف في حلقي. I was aware of dryness of my mouth.	0	1	2	3
لم يبدو لي أن بإمكاني الإحساس بمشاعر إيجابية على الإطلاق. I couldn’t seem to experience any positive feeling at all.	0	1	2	3
شعرت بصعوبة في التنفس (شدة التنفس السريع، اللهثان بدون القيام بمجهود جسدي مثلًا). I experienced breathing difficulty (e.g., excessively rapid breathing, breathlessness in the absence of physical exertion).	0	1	2	3
وجدت صعوبة بأخذ المبادرة بعمل الأشياء. I found it difficult to work up the initiative to do things.	0	1	2	3
كنت أميل إلى ردة فعل مفرطة للظروف والأحداث. I tended to over-react to situations.	0	1	2	3
شعرت برجفة (باليدين مثلًا). I experienced trembling (e.g., in the hands).	0	1	2	3
شعرت بأنني أستهلك الكثير في الطاقة العصبية (شعرت بأنني أستهلك الكثير من قدرتي على تحمل التوتر العصبي). I felt that I was using a lot of nervous energy.	0	1	2	3
كنت خائفًا من مواقف قد أفقد فيها السيطرة على أعصابي واسبب إحراجًا لنفسي. I was worried about situations in which I might panic and make a fool of myself.	0	1	2	3
شعرت بأن ليس لدي أي شيء أتطلع إليه. I felt that I had nothing to look forward to.	0	1	2	3
شعرت بأنني مضطرب ومنزعج. I found myself getting agitated.	0	1	2	3
أجد صعوبة في الاسترخاء. I found it difficult to relax.	0	1	2	3
شعرت بالحزن والغم. I felt downhearted and blue.	0	1	2	3
كنت لا أستطع تحمل أي شيء يحول بيني وبين ما أرغب في القيام به. I was intolerant of anything that kept me from getting on with what I was doing.	0	1	2	3
شعرت بأنني على وشك الوقوع في حالة من الرعب المفاجئ بدون سبب. I felt I was close to panic.	0	1	2	3
فقدت الشعور بالحماس لأي شيء. I was unable to become enthusiastic about anything.	0	1	2	3
شعرت بأن قيمتي قليلة كشخص. I felt I wasn’t worth much as a person.	0	1	2	3
شعرت بأنني أميل إلى الغيظ بسرعة. I felt that I was rather touchy.	0	1	2	3
شعرت بضربات قلبي بدون مجهود جسدي (زيادة في معدل الدقات، أو غياب دقة قلب مثلًا). I was aware of the action of my heart in the absence of physical exertion (e.g., sense of heart rate increase, heart missing a beat).	0	1	2	3
شعرت بالخوف بدون أي سبب وجيه. I felt scared without any good reason.	0	1	2	3
شعرت بأن الحياة ليس لها معنى. I felt that life was meaningless.	0	1	2	3
